# {Bis[2-(dicyclo­hexyl­phosphino)phen­yl]methyl­silyl-κ^3^ *P*,*Si*,*P*′}chloridoplatinum(II)

**DOI:** 10.1107/S1600536808028857

**Published:** 2008-09-17

**Authors:** Yong-Hua Li, Yuan Zhang, Min-Min Zhao, Xian Li

**Affiliations:** a Ordered Matter Science Research Center, College of Chemistry and Chemical Engineering, Southeast University, Nanjing 211189, People’s Republic of China

## Abstract

In the title compound, [Pt(C_37_H_55_P_2_Si)Cl], prepared from MeSiH[(cy)_2_PC_6_H_4_]_2_ and [Pt(cod)Cl_2_] (cy = cyclo­hexyl; cod = cyclo­octa-1,5-diene), the Pt^II^ atom is coordinated by two P atoms, one Si atom and one Cl atom in a distorted square-planar geometry. The two P atoms are in a *trans* arrangement and the four cyclo­hexane rings adopt a chair conformation.

## Related literature

For related literature, see: van der Boom & Milstein (2003 ▶); Brost *et al.* (1997 ▶); Moulton & Shaw (1976 ▶).
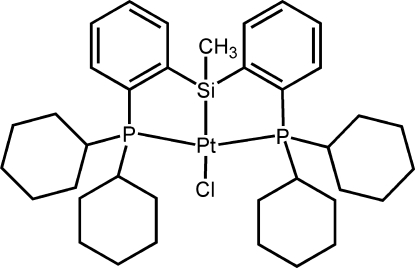


## Experimental

### 

#### Crystal data

[Pt(C_37_H_55_P_2_Si)Cl]
*M* *_r_* = 820.38
Monoclinic, 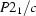

*a* = 13.104 (3) Å
*b* = 16.579 (3) Å
*c* = 17.770 (4) Å
β = 108.97 (3)°
*V* = 3650.9 (15) Å^3^
*Z* = 4
Mo *K*α radiation
μ = 4.06 mm^−1^
*T* = 153 (2) K
0.48 × 0.40 × 0.40 mm

#### Data collection

Bruker SMART APEX CCD area-detector diffractometer
Absorption correction: multi-scan (*SADABS*; Sheldrick, 1996 ▶) *T*_min_ = 0.120, *T*_max_ = 0.200
36373 measured reflections
8374 independent reflections
7059 reflections with *I* > 2σ(*I*)
*R*_int_ = 0.035

#### Refinement

*R*[*F* ^2^ > 2σ(*F* ^2^)] = 0.020
*wR*(*F* ^2^) = 0.047
*S* = 0.97
8374 reflections
380 parameters
H-atom parameters constrained
Δρ_max_ = 1.00 e Å^−3^
Δρ_min_ = −0.55 e Å^−3^

### 

Data collection: *SMART* (Bruker, 2007 ▶); cell refinement: *SAINT* (Bruker, 2007 ▶); data reduction: *SAINT*; program(s) used to solve structure: *SHELXS97* (Sheldrick, 2008 ▶); program(s) used to refine structure: *SHELXL97* (Sheldrick, 2008 ▶); molecular graphics: *SHELXTL* (Sheldrick, 2008 ▶); software used to prepare material for publication: *SHELXTL*.

## Supplementary Material

Crystal structure: contains datablocks I, global. DOI: 10.1107/S1600536808028857/hy2153sup1.cif

Structure factors: contains datablocks I. DOI: 10.1107/S1600536808028857/hy2153Isup2.hkl

Additional supplementary materials:  crystallographic information; 3D view; checkCIF report

## Figures and Tables

**Table:** 

Pt1—Si1	2.2790 (7)
Pt1—P1	2.2925 (8)
Pt1—P2	2.2929 (7)
Pt1—Cl1	2.4597 (7)

**Table:** 

Si1—Pt1—P1	84.89 (3)
Si1—Pt1—P2	84.57 (3)
P1—Pt1—P2	162.15 (2)
Si1—Pt1—Cl1	178.03 (2)
P1—Pt1—Cl1	93.68 (3)
P2—Pt1—Cl1	97.15 (3)

**Fig. 1.**
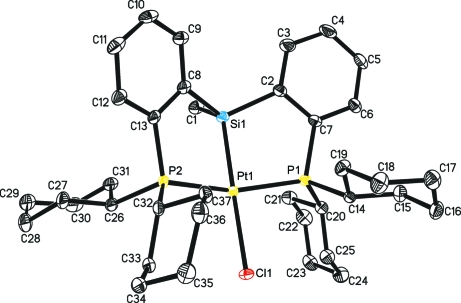
Molecular structure of the title compound. Displacement ellipsoids are drawn at the 30% probability level. H atoms have been omitted for clarity.

## supplementary crystallographic information

### Comment

Pincers ligands incorporating two phosphine arms and a central donor site have attracted a substantial amount of interest since the initial investigations of "PCP" ligands by Moulton & Shaw (1976). Several variations of the central donor atom have been explored (Boom & Milstein, 2003). However, the "PSiP" pincers-like transition metal complexes have rarely been reported. We report here the synthesis and structure of a new Pt(η^3^-PSiP) complex. The molecular structure of the title compound is shown in Fig. 1.

The pincers-like title compound contains two stable five-membered cyclometalated rings with the P—Pt—Si angles of 84.89 (3) and 84.57 (3) ° (Table 1). The Pt atom is coordinated by two P atoms, one Si atom and one Cl atom in a distorted square-planar geometry. The bond distances of Pt—Si and Pt—Cl are 2.2790 (7) and 2.4597 (7) Å, respectively, which are similar to the other Pt analogue with pincers-like tridentate PSiP ligand, Pt[SiMe(CH_2_CH_2_CH_2_PPh_2_)_2_]Cl (Brost *et al.*, 1997). The two P donor atoms are in a *trans* arrangement with a P—Pt—P angle of 162.15 (2)°. The four cyclohexane rings adopt the chair conformation.

### Experimental

Dropwise addition of a solution of MeSiH[(cy)_2_PC_6_H_4_]_2_ (0.124 g, 0.21 mmol) (cy = cyclohexyl) in dry THF (5 ml) to a solution of [Pt(cod)Cl_2_] (0.079 g, 0.21 mmol) (cod = cycloocta-1,5-diene) in a mixture of THF (7 ml) and NEt_3_ (1 ml) resulted in rapid formation of a white precipitate. Removal of the volatiles left solid material, which gave the product after thorough washing (yield 78%, 0.134 g). Colorless crystals suitable for X-ray diffraction were obtained by slow evaporation of a benzene solution (5 ml) of the compound (0.028 g) after 2 d.

### Refinement

H atoms were positioned geometrically and refined using a riding model, with C—H = 0.93 (aromatic), 0.98 (CH), 0.97 (CH_2_), 0.96 (CH_3_) Å and *U*_iso_(H) = 1.2(or 1.5 for methyl)*U*_eq_(C).

### Crystal data

**Table:**

[Pt(C_37_H_55_P_2_Si)Cl]	*F*(000) = 1664
*M**_r_* = 820.38	*D*_x_ = 1.493 Mg m^−^^3^
Monoclinic, *P*2_1_/*c*	Mo *K*α radiation, λ = 0.71073 Å
Hall symbol: -P 2ybc	Cell parameters from 8404 reflections
*a* = 13.104 (3) Å	θ = 3.0–27.5°
*b* = 16.579 (3) Å	µ = 4.06 mm^−^^1^
*c* = 17.770 (4) Å	*T* = 153 K
β = 108.97 (3)°	Block, colorless
*V* = 3650.9 (15) Å^3^	0.48 × 0.40 × 0.40 mm
*Z* = 4	

### Data collection

**Table:**

Bruker SMART APEX CCD area-detector diffractometer	8374 independent reflections
Radiation source: fine-focus sealed tube	7059 reflections with *I* > 2σ(*I*)
graphite	*R*_int_ = 0.035
φ and ω scans	θ_max_ = 27.5°, θ_min_ = 1.6°
Absorption correction: multi-scan (SADABS; Sheldrick, 1996)	*h* = −16→17
*T*_min_ = 0.120, *T*_max_ = 0.200	*k* = −21→21
36373 measured reflections	*l* = −23→23

### Refinement

**Table:**

Refinement on *F*^2^	Primary atom site location: structure-invariant direct methods
Least-squares matrix: full	Secondary atom site location: difference Fourier map
*R*[*F*^2^ > 2σ(*F*^2^)] = 0.020	Hydrogen site location: inferred from neighbouring sites
*wR*(*F*^2^) = 0.047	H-atom parameters constrained
*S* = 0.97	*w* = 1/[σ^2^(*F*_o_^2^) + (0.0241*P*)^2^] where *P* = (*F*_o_^2^ + 2*F*_c_^2^)/3
8374 reflections	(Δ/σ)_max_ = 0.003
380 parameters	Δρ_max_ = 1.00 e Å^−^^3^
0 restraints	Δρ_min_ = −0.55 e Å^−^^3^

### Fractional atomic coordinates and isotropic or equivalent isotropic displacement parameters (Å^2^)

**Table:**

	*x*	*y*	*z*	*U*_iso_*/*U*_eq_	
Si1	0.79709 (6)	0.29114 (4)	0.75245 (4)	0.02238 (15)	
C1	0.8794 (2)	0.38510 (15)	0.79101 (16)	0.0312 (6)	
H9	0.9530	0.3703	0.8182	0.047*	
H10	0.8507	0.4127	0.8272	0.047*	
H11	0.8762	0.4200	0.7472	0.047*	
C2	0.6609 (2)	0.33576 (15)	0.69588 (15)	0.0258 (6)	
C3	0.6388 (2)	0.38438 (17)	0.62824 (16)	0.0383 (7)	
H1	0.6903	0.3894	0.6028	0.046*	
C4	0.5422 (3)	0.42510 (19)	0.59835 (18)	0.0459 (8)	
H2	0.5281	0.4550	0.5518	0.055*	
C5	0.4668 (2)	0.42180 (17)	0.63697 (16)	0.0371 (7)	
H3	0.4031	0.4511	0.6178	0.045*	
C6	0.4859 (2)	0.37465 (15)	0.70450 (15)	0.0291 (6)	
H4	0.4351	0.3724	0.7307	0.035*	
C7	0.5818 (2)	0.33037 (15)	0.73334 (14)	0.0236 (5)	
C8	0.8674 (2)	0.23542 (15)	0.69018 (14)	0.0242 (5)	
C9	0.8662 (2)	0.25752 (16)	0.61413 (15)	0.0284 (6)	
H5	0.8272	0.3027	0.5900	0.034*	
C10	0.9217 (2)	0.21384 (17)	0.57387 (16)	0.0341 (7)	
H6	0.9181	0.2288	0.5226	0.041*	
C11	0.9826 (2)	0.14780 (19)	0.60967 (16)	0.0367 (7)	
H7	1.0214	0.1190	0.5831	0.044*	
C12	0.9855 (2)	0.12458 (17)	0.68555 (16)	0.0313 (6)	
H8	1.0269	0.0804	0.7098	0.038*	
C13	0.92692 (19)	0.16711 (16)	0.72559 (14)	0.0236 (5)	
P1	0.61006 (5)	0.26215 (4)	0.81899 (4)	0.01969 (13)	
P2	0.91721 (5)	0.13477 (4)	0.82203 (4)	0.02147 (14)	
Pt1	0.773705 (7)	0.200163 (5)	0.841596 (5)	0.01795 (3)	
Cl1	0.74599 (5)	0.10572 (4)	0.93994 (3)	0.02439 (13)	
C14	0.50326 (19)	0.18463 (14)	0.78938 (14)	0.0217 (5)	
H52	0.5189	0.1456	0.8331	0.026*	
C15	0.3878 (2)	0.21183 (16)	0.77279 (18)	0.0338 (6)	
H12	0.3691	0.2507	0.7297	0.041*	
H13	0.3810	0.2382	0.8197	0.041*	
C16	0.3096 (2)	0.14051 (18)	0.75032 (18)	0.0387 (7)	
H14	0.3256	0.1030	0.7945	0.046*	
H15	0.2363	0.1597	0.7395	0.046*	
C17	0.3191 (2)	0.09773 (18)	0.67749 (17)	0.0400 (7)	
H16	0.2704	0.0519	0.6648	0.048*	
H17	0.2984	0.1343	0.6324	0.048*	
C18	0.4322 (2)	0.06942 (18)	0.69221 (19)	0.0434 (8)	
H18	0.4378	0.0447	0.6442	0.052*	
H19	0.4502	0.0290	0.7339	0.052*	
C19	0.5119 (2)	0.13911 (17)	0.71671 (16)	0.0317 (6)	
H20	0.4987	0.1764	0.6725	0.038*	
H21	0.5846	0.1183	0.7287	0.038*	
C20	0.5814 (2)	0.32223 (15)	0.89640 (15)	0.0262 (6)	
H53	0.5119	0.3491	0.8720	0.031*	
C21	0.6675 (2)	0.38825 (16)	0.92785 (16)	0.0315 (6)	
H22	0.6709	0.4219	0.8841	0.038*	
H23	0.7376	0.3635	0.9519	0.038*	
C22	0.6391 (3)	0.44040 (17)	0.99003 (17)	0.0410 (7)	
H24	0.6953	0.4803	1.0112	0.049*	
H25	0.5720	0.4688	0.9645	0.049*	
C23	0.6275 (2)	0.39036 (18)	1.05693 (16)	0.0388 (7)	
H26	0.6051	0.4245	1.0930	0.047*	
H27	0.6969	0.3670	1.0864	0.047*	
C24	0.5450 (3)	0.32308 (18)	1.02620 (17)	0.0395 (7)	
H28	0.4740	0.3466	1.0025	0.047*	
H29	0.5431	0.2897	1.0705	0.047*	
C25	0.5719 (2)	0.27061 (17)	0.96458 (16)	0.0335 (6)	
H30	0.6394	0.2425	0.9895	0.040*	
H31	0.5157	0.2306	0.9441	0.040*	
C26	1.0505 (2)	0.14993 (17)	0.89855 (15)	0.0288 (6)	
H54	1.0436	0.1302	0.9486	0.035*	
C27	1.1442 (2)	0.1034 (2)	0.88627 (16)	0.0391 (7)	
H32	1.1545	0.1208	0.8371	0.047*	
H33	1.1272	0.0463	0.8817	0.047*	
C28	1.2483 (2)	0.1173 (2)	0.95581 (19)	0.0495 (9)	
H34	1.2405	0.0942	1.0038	0.059*	
H35	1.3074	0.0897	0.9450	0.059*	
C29	1.2751 (2)	0.2056 (2)	0.96936 (19)	0.0505 (9)	
H36	1.2933	0.2268	0.9245	0.061*	
H37	1.3378	0.2118	1.0166	0.061*	
C30	1.1819 (2)	0.2537 (2)	0.97950 (17)	0.0452 (8)	
H38	1.1999	0.3106	0.9826	0.054*	
H39	1.1713	0.2383	1.0291	0.054*	
C31	1.0772 (2)	0.24003 (18)	0.91103 (15)	0.0327 (6)	
H40	1.0840	0.2627	0.8626	0.039*	
H41	1.0186	0.2677	0.9224	0.039*	
C32	0.90420 (19)	0.02456 (15)	0.80991 (14)	0.0239 (5)	
H55	0.9576	0.0076	0.7852	0.029*	
C33	0.9287 (2)	−0.02323 (15)	0.88762 (15)	0.0285 (6)	
H42	0.9990	−0.0082	0.9239	0.034*	
H43	0.8750	−0.0113	0.9129	0.034*	
C34	0.9270 (2)	−0.11266 (17)	0.86878 (17)	0.0351 (7)	
H44	0.9416	−0.1433	0.9177	0.042*	
H45	0.9835	−0.1245	0.8463	0.042*	
C35	0.8189 (2)	−0.13878 (17)	0.81058 (18)	0.0391 (7)	
H46	0.7635	−0.1328	0.8354	0.047*	
H47	0.8225	−0.1953	0.7976	0.047*	
C36	0.7884 (2)	−0.08889 (16)	0.73421 (17)	0.0365 (7)	
H48	0.8372	−0.1015	0.7050	0.044*	
H49	0.7159	−0.1031	0.7010	0.044*	
C37	0.7934 (2)	0.00123 (15)	0.75192 (15)	0.0275 (6)	
H50	0.7379	0.0153	0.7748	0.033*	
H51	0.7797	0.0312	0.7028	0.033*	

### Atomic displacement parameters (Å^2^)

**Table:**

	*U*^11^	*U*^22^	*U*^33^	*U*^12^	*U*^13^	*U*^23^
Si1	0.0229 (4)	0.0234 (4)	0.0197 (3)	−0.0075 (3)	0.0055 (3)	0.0016 (3)
C1	0.0347 (16)	0.0268 (14)	0.0318 (14)	−0.0124 (12)	0.0105 (12)	−0.0021 (12)
C2	0.0263 (14)	0.0236 (13)	0.0234 (13)	−0.0072 (11)	0.0025 (11)	0.0038 (11)
C3	0.0382 (17)	0.0409 (17)	0.0336 (15)	−0.0076 (14)	0.0087 (13)	0.0117 (13)
C4	0.0450 (19)	0.0461 (19)	0.0362 (17)	−0.0073 (15)	−0.0012 (15)	0.0230 (14)
C5	0.0279 (16)	0.0321 (16)	0.0380 (16)	−0.0043 (12)	−0.0076 (13)	0.0108 (13)
C6	0.0250 (14)	0.0227 (14)	0.0325 (14)	−0.0067 (11)	−0.0005 (12)	0.0021 (11)
C7	0.0269 (14)	0.0192 (12)	0.0198 (12)	−0.0066 (11)	0.0008 (11)	0.0014 (10)
C8	0.0218 (13)	0.0264 (13)	0.0233 (13)	−0.0145 (11)	0.0057 (11)	−0.0043 (11)
C9	0.0298 (15)	0.0296 (15)	0.0245 (13)	−0.0153 (12)	0.0068 (12)	−0.0017 (11)
C10	0.0440 (18)	0.0397 (17)	0.0207 (13)	−0.0213 (14)	0.0135 (13)	−0.0062 (12)
C11	0.0377 (17)	0.0468 (18)	0.0320 (15)	−0.0157 (14)	0.0200 (14)	−0.0129 (14)
C12	0.0265 (15)	0.0388 (16)	0.0294 (14)	−0.0083 (12)	0.0101 (12)	−0.0049 (12)
C13	0.0180 (13)	0.0298 (14)	0.0222 (12)	−0.0105 (11)	0.0054 (10)	−0.0036 (11)
P1	0.0205 (3)	0.0193 (3)	0.0179 (3)	−0.0017 (3)	0.0045 (3)	0.0005 (2)
P2	0.0151 (3)	0.0279 (3)	0.0203 (3)	−0.0040 (3)	0.0043 (3)	0.0009 (3)
Pt1	0.01686 (5)	0.02063 (5)	0.01516 (5)	−0.00361 (4)	0.00354 (3)	0.00086 (4)
Cl1	0.0328 (3)	0.0227 (3)	0.0213 (3)	0.0015 (3)	0.0137 (3)	0.0035 (2)
C14	0.0198 (13)	0.0233 (13)	0.0198 (12)	−0.0034 (10)	0.0033 (10)	0.0037 (10)
C15	0.0257 (15)	0.0345 (16)	0.0399 (16)	−0.0010 (12)	0.0086 (13)	−0.0012 (13)
C16	0.0266 (15)	0.0383 (17)	0.0513 (18)	−0.0048 (13)	0.0128 (14)	0.0040 (14)
C17	0.0362 (17)	0.0354 (16)	0.0436 (17)	−0.0076 (14)	0.0063 (14)	−0.0053 (14)
C18	0.0410 (19)	0.0401 (18)	0.0491 (19)	−0.0130 (14)	0.0148 (15)	−0.0134 (15)
C19	0.0322 (16)	0.0340 (15)	0.0297 (14)	−0.0127 (12)	0.0113 (12)	−0.0088 (12)
C20	0.0276 (14)	0.0246 (13)	0.0255 (13)	0.0013 (11)	0.0077 (11)	−0.0014 (11)
C21	0.0408 (17)	0.0240 (14)	0.0288 (14)	−0.0044 (12)	0.0100 (13)	−0.0043 (11)
C22	0.048 (2)	0.0273 (15)	0.0414 (17)	−0.0023 (14)	0.0053 (15)	−0.0083 (13)
C23	0.0417 (18)	0.0408 (17)	0.0291 (15)	0.0085 (14)	0.0050 (13)	−0.0085 (13)
C24	0.0437 (19)	0.0451 (18)	0.0312 (15)	0.0015 (14)	0.0141 (14)	−0.0079 (13)
C25	0.0352 (16)	0.0336 (15)	0.0303 (14)	−0.0012 (13)	0.0089 (13)	−0.0041 (12)
C26	0.0182 (13)	0.0439 (17)	0.0217 (13)	−0.0082 (12)	0.0031 (11)	0.0022 (12)
C27	0.0186 (14)	0.061 (2)	0.0355 (16)	−0.0029 (14)	0.0061 (12)	0.0026 (15)
C28	0.0167 (14)	0.078 (3)	0.0464 (19)	−0.0048 (16)	0.0004 (14)	0.0082 (17)
C29	0.0211 (15)	0.084 (3)	0.0388 (17)	−0.0208 (16)	−0.0005 (13)	0.0099 (17)
C30	0.0386 (18)	0.059 (2)	0.0303 (15)	−0.0290 (16)	0.0009 (14)	0.0019 (15)
C31	0.0265 (15)	0.0449 (17)	0.0237 (13)	−0.0166 (13)	0.0043 (12)	0.0004 (12)
C32	0.0157 (12)	0.0263 (13)	0.0288 (13)	0.0015 (10)	0.0059 (11)	0.0013 (11)
C33	0.0208 (14)	0.0318 (15)	0.0330 (14)	0.0051 (11)	0.0087 (12)	0.0073 (12)
C34	0.0310 (16)	0.0359 (16)	0.0424 (16)	0.0111 (13)	0.0174 (13)	0.0140 (13)
C35	0.0387 (18)	0.0237 (15)	0.058 (2)	0.0011 (13)	0.0205 (16)	0.0021 (14)
C36	0.0301 (16)	0.0315 (16)	0.0474 (18)	−0.0021 (13)	0.0118 (14)	−0.0067 (13)
C37	0.0195 (13)	0.0262 (14)	0.0327 (14)	−0.0001 (11)	0.0030 (11)	0.0009 (12)

### Geometric parameters (Å, °)

**Table:**

Si1—C2	1.891 (3)	C19—H21	0.9700
Si1—C1	1.892 (3)	C20—C25	1.521 (4)
Si1—C8	1.894 (3)	C20—C21	1.540 (4)
Pt1—Si1	2.2790 (7)	C20—H53	0.9800
C1—H9	0.9600	C21—C22	1.541 (4)
C1—H10	0.9600	C21—H22	0.9700
C1—H11	0.9600	C21—H23	0.9700
C2—C3	1.397 (4)	C22—C23	1.497 (4)
C2—C7	1.405 (4)	C22—H24	0.9700
C3—C4	1.379 (4)	C22—H25	0.9700
C3—H1	0.9300	C23—C24	1.526 (4)
C4—C5	1.376 (4)	C23—H26	0.9700
C4—H2	0.9300	C23—H27	0.9700
C5—C6	1.385 (4)	C24—C25	1.527 (4)
C5—H3	0.9300	C24—H28	0.9700
C6—C7	1.401 (4)	C24—H29	0.9700
C6—H4	0.9300	C25—H30	0.9700
C7—P1	1.835 (2)	C25—H31	0.9700
C8—C9	1.396 (3)	C26—C27	1.524 (4)
C8—C13	1.403 (4)	C26—C31	1.534 (4)
C9—C10	1.379 (4)	C26—H54	0.9800
C9—H5	0.9300	C27—C28	1.532 (4)
C10—C11	1.382 (4)	C27—H32	0.9700
C10—H6	0.9300	C27—H33	0.9700
C11—C12	1.391 (4)	C28—C29	1.507 (4)
C11—H7	0.9300	C28—H34	0.9700
C12—C13	1.396 (4)	C28—H35	0.9700
C12—H8	0.9300	C29—C30	1.518 (5)
C13—P2	1.839 (2)	C29—H36	0.9700
P1—C20	1.833 (3)	C29—H37	0.9700
P1—C14	1.846 (2)	C30—C31	1.526 (4)
Pt1—P1	2.2925 (8)	C30—H38	0.9700
P2—C32	1.841 (3)	C30—H39	0.9700
P2—C26	1.849 (3)	C31—H40	0.9700
Pt1—P2	2.2929 (7)	C31—H41	0.9700
Pt1—Cl1	2.4597 (7)	C32—C37	1.532 (3)
C14—C15	1.513 (4)	C32—C33	1.533 (3)
C14—C19	1.531 (3)	C32—H55	0.9800
C14—H52	0.9800	C33—C34	1.519 (4)
C15—C16	1.530 (4)	C33—H42	0.9700
C15—H12	0.9700	C33—H43	0.9700
C15—H13	0.9700	C34—C35	1.520 (4)
C16—C17	1.516 (4)	C34—H44	0.9700
C16—H14	0.9700	C34—H45	0.9700
C16—H15	0.9700	C35—C36	1.527 (4)
C17—C18	1.495 (4)	C35—H46	0.9700
C17—H16	0.9700	C35—H47	0.9700
C17—H17	0.9700	C36—C37	1.524 (4)
C18—C19	1.523 (4)	C36—H48	0.9700
C18—H18	0.9700	C36—H49	0.9700
C18—H19	0.9700	C37—H50	0.9700
C19—H20	0.9700	C37—H51	0.9700
			
C2—Si1—C1	101.47 (12)	C25—C20—H53	107.6
C2—Si1—C8	115.74 (12)	C21—C20—H53	107.6
C1—Si1—C8	106.56 (12)	P1—C20—H53	107.6
C2—Si1—Pt1	108.15 (8)	C20—C21—C22	109.9 (2)
C1—Si1—Pt1	118.87 (9)	C20—C21—H22	109.7
C8—Si1—Pt1	106.50 (8)	C22—C21—H22	109.7
Si1—C1—H9	109.5	C20—C21—H23	109.7
Si1—C1—H10	109.5	C22—C21—H23	109.7
H9—C1—H10	109.5	H22—C21—H23	108.2
Si1—C1—H11	109.5	C23—C22—C21	111.6 (2)
H9—C1—H11	109.5	C23—C22—H24	109.3
H10—C1—H11	109.5	C21—C22—H24	109.3
C3—C2—C7	117.8 (3)	C23—C22—H25	109.3
C3—C2—Si1	125.4 (2)	C21—C22—H25	109.3
C7—C2—Si1	115.93 (18)	H24—C22—H25	108.0
C4—C3—C2	121.4 (3)	C22—C23—C24	111.3 (2)
C4—C3—H1	119.3	C22—C23—H26	109.4
C2—C3—H1	119.3	C24—C23—H26	109.4
C5—C4—C3	120.4 (3)	C22—C23—H27	109.4
C5—C4—H2	119.8	C24—C23—H27	109.4
C3—C4—H2	119.8	H26—C23—H27	108.0
C4—C5—C6	119.8 (3)	C23—C24—C25	111.8 (3)
C4—C5—H3	120.1	C23—C24—H28	109.3
C6—C5—H3	120.1	C25—C24—H28	109.3
C5—C6—C7	120.1 (3)	C23—C24—H29	109.3
C5—C6—H4	120.0	C25—C24—H29	109.3
C7—C6—H4	120.0	H28—C24—H29	107.9
C6—C7—C2	120.4 (2)	C20—C25—C24	110.4 (2)
C6—C7—P1	122.8 (2)	C20—C25—H30	109.6
C2—C7—P1	116.83 (19)	C24—C25—H30	109.6
C9—C8—C13	118.4 (2)	C20—C25—H31	109.6
C9—C8—Si1	125.7 (2)	C24—C25—H31	109.6
C13—C8—Si1	115.90 (18)	H30—C25—H31	108.1
C10—C9—C8	121.5 (3)	C27—C26—C31	110.9 (2)
C10—C9—H5	119.2	C27—C26—P2	115.99 (19)
C8—C9—H5	119.2	C31—C26—P2	110.80 (18)
C9—C10—C11	120.0 (2)	C27—C26—H54	106.1
C9—C10—H6	120.0	C31—C26—H54	106.1
C11—C10—H6	120.0	P2—C26—H54	106.1
C10—C11—C12	119.7 (3)	C26—C27—C28	110.9 (2)
C10—C11—H7	120.2	C26—C27—H32	109.5
C12—C11—H7	120.2	C28—C27—H32	109.5
C11—C12—C13	120.5 (3)	C26—C27—H33	109.5
C11—C12—H8	119.7	C28—C27—H33	109.5
C13—C12—H8	119.7	H32—C27—H33	108.0
C12—C13—C8	119.8 (2)	C29—C28—C27	112.0 (3)
C12—C13—P2	122.9 (2)	C29—C28—H34	109.2
C8—C13—P2	117.15 (19)	C27—C28—H34	109.2
C20—P1—C7	104.61 (12)	C29—C28—H35	109.2
C20—P1—C14	105.71 (12)	C27—C28—H35	109.2
C7—P1—C14	105.24 (11)	H34—C28—H35	107.9
C20—P1—Pt1	121.34 (9)	C28—C29—C30	112.0 (2)
C7—P1—Pt1	110.28 (9)	C28—C29—H36	109.2
C14—P1—Pt1	108.49 (8)	C30—C29—H36	109.2
C13—P2—C32	102.25 (12)	C28—C29—H37	109.2
C13—P2—C26	108.10 (11)	C30—C29—H37	109.2
C32—P2—C26	104.49 (12)	H36—C29—H37	107.9
C13—P2—Pt1	108.18 (9)	C29—C30—C31	112.2 (3)
C32—P2—Pt1	115.95 (8)	C29—C30—H38	109.2
C26—P2—Pt1	116.71 (9)	C31—C30—H38	109.2
Si1—Pt1—P1	84.89 (3)	C29—C30—H39	109.2
Si1—Pt1—P2	84.57 (3)	C31—C30—H39	109.2
P1—Pt1—P2	162.15 (2)	H38—C30—H39	107.9
Si1—Pt1—Cl1	178.03 (2)	C30—C31—C26	111.3 (2)
P1—Pt1—Cl1	93.68 (3)	C30—C31—H40	109.4
P2—Pt1—Cl1	97.15 (3)	C26—C31—H40	109.4
C15—C14—C19	109.0 (2)	C30—C31—H41	109.4
C15—C14—P1	117.74 (18)	C26—C31—H41	109.4
C19—C14—P1	109.06 (17)	H40—C31—H41	108.0
C15—C14—H52	106.8	C37—C32—C33	110.5 (2)
C19—C14—H52	106.8	C37—C32—P2	111.14 (17)
P1—C14—H52	106.8	C33—C32—P2	115.04 (17)
C14—C15—C16	111.3 (2)	C37—C32—H55	106.5
C14—C15—H12	109.4	C33—C32—H55	106.5
C16—C15—H12	109.4	P2—C32—H55	106.5
C14—C15—H13	109.4	C34—C33—C32	108.8 (2)
C16—C15—H13	109.4	C34—C33—H42	109.9
H12—C15—H13	108.0	C32—C33—H42	109.9
C17—C16—C15	110.5 (2)	C34—C33—H43	109.9
C17—C16—H14	109.5	C32—C33—H43	109.9
C15—C16—H14	109.5	H42—C33—H43	108.3
C17—C16—H15	109.5	C33—C34—C35	111.8 (2)
C15—C16—H15	109.5	C33—C34—H44	109.2
H14—C16—H15	108.1	C35—C34—H44	109.2
C18—C17—C16	110.5 (2)	C33—C34—H45	109.2
C18—C17—H16	109.6	C35—C34—H45	109.2
C16—C17—H16	109.6	H44—C34—H45	107.9
C18—C17—H17	109.6	C34—C35—C36	111.6 (2)
C16—C17—H17	109.6	C34—C35—H46	109.3
H16—C17—H17	108.1	C36—C35—H46	109.3
C17—C18—C19	111.1 (3)	C34—C35—H47	109.3
C17—C18—H18	109.4	C36—C35—H47	109.3
C19—C18—H18	109.4	H46—C35—H47	108.0
C17—C18—H19	109.4	C37—C36—C35	111.5 (2)
C19—C18—H19	109.4	C37—C36—H48	109.3
H18—C18—H19	108.0	C35—C36—H48	109.3
C18—C19—C14	112.5 (2)	C37—C36—H49	109.3
C18—C19—H20	109.1	C35—C36—H49	109.3
C14—C19—H20	109.1	H48—C36—H49	108.0
C18—C19—H21	109.1	C36—C37—C32	110.7 (2)
C14—C19—H21	109.1	C36—C37—H50	109.5
H20—C19—H21	107.8	C32—C37—H50	109.5
C25—C20—C21	110.5 (2)	C36—C37—H51	109.5
C25—C20—P1	112.44 (18)	C32—C37—H51	109.5
C21—C20—P1	110.72 (18)	H50—C37—H51	108.1
			
C1—Si1—C2—C3	64.5 (3)	C26—P2—Pt1—Si1	100.07 (10)
C8—Si1—C2—C3	−50.4 (3)	C13—P2—Pt1—P1	32.02 (12)
Pt1—Si1—C2—C3	−169.7 (2)	C32—P2—Pt1—P1	−82.07 (12)
C1—Si1—C2—C7	−104.1 (2)	C26—P2—Pt1—P1	154.11 (11)
C8—Si1—C2—C7	140.99 (19)	C13—P2—Pt1—Cl1	158.94 (8)
Pt1—Si1—C2—C7	21.7 (2)	C32—P2—Pt1—Cl1	44.85 (9)
C7—C2—C3—C4	−0.8 (4)	C26—P2—Pt1—Cl1	−78.97 (10)
Si1—C2—C3—C4	−169.2 (2)	C20—P1—C14—C15	47.3 (2)
C2—C3—C4—C5	3.1 (5)	C7—P1—C14—C15	−63.1 (2)
C3—C4—C5—C6	−2.6 (5)	Pt1—P1—C14—C15	178.91 (17)
C4—C5—C6—C7	−0.1 (4)	C20—P1—C14—C19	172.15 (18)
C5—C6—C7—C2	2.4 (4)	C7—P1—C14—C19	61.8 (2)
C5—C6—C7—P1	−176.5 (2)	Pt1—P1—C14—C19	−56.25 (18)
C3—C2—C7—C6	−2.0 (4)	C19—C14—C15—C16	56.0 (3)
Si1—C2—C7—C6	167.58 (19)	P1—C14—C15—C16	−179.13 (19)
C3—C2—C7—P1	177.00 (19)	C14—C15—C16—C17	−58.6 (3)
Si1—C2—C7—P1	−13.5 (3)	C15—C16—C17—C18	57.8 (3)
C2—Si1—C8—C9	37.3 (2)	C16—C17—C18—C19	−56.2 (3)
C1—Si1—C8—C9	−74.6 (2)	C17—C18—C19—C14	55.6 (3)
Pt1—Si1—C8—C9	157.52 (19)	C15—C14—C19—C18	−54.8 (3)
C2—Si1—C8—C13	−144.02 (18)	P1—C14—C19—C18	175.4 (2)
C1—Si1—C8—C13	104.1 (2)	C7—P1—C20—C25	165.15 (19)
Pt1—Si1—C8—C13	−23.8 (2)	C14—P1—C20—C25	54.3 (2)
C13—C8—C9—C10	0.1 (4)	Pt1—P1—C20—C25	−69.5 (2)
Si1—C8—C9—C10	178.77 (19)	C7—P1—C20—C21	−70.7 (2)
C8—C9—C10—C11	−1.8 (4)	C14—P1—C20—C21	178.52 (17)
C9—C10—C11—C12	1.5 (4)	Pt1—P1—C20—C21	54.7 (2)
C10—C11—C12—C13	0.4 (4)	C25—C20—C21—C22	−57.3 (3)
C11—C12—C13—C8	−2.1 (4)	P1—C20—C21—C22	177.45 (18)
C11—C12—C13—P2	174.4 (2)	C20—C21—C22—C23	56.5 (3)
C9—C8—C13—C12	1.8 (4)	C21—C22—C23—C24	−55.3 (3)
Si1—C8—C13—C12	−176.97 (19)	C22—C23—C24—C25	55.1 (3)
C9—C8—C13—P2	−174.89 (18)	C21—C20—C25—C24	57.2 (3)
Si1—C8—C13—P2	6.3 (3)	P1—C20—C25—C24	−178.5 (2)
C6—C7—P1—C20	−49.8 (2)	C23—C24—C25—C20	−55.9 (3)
C2—C7—P1—C20	131.3 (2)	C13—P2—C26—C27	−60.2 (2)
C6—C7—P1—C14	61.4 (2)	C32—P2—C26—C27	48.2 (2)
C2—C7—P1—C14	−117.6 (2)	Pt1—P2—C26—C27	177.69 (17)
C6—C7—P1—Pt1	178.19 (18)	C13—P2—C26—C31	67.4 (2)
C2—C7—P1—Pt1	−0.7 (2)	C32—P2—C26—C31	175.79 (17)
C12—C13—P2—C32	−39.7 (2)	Pt1—P2—C26—C31	−54.71 (19)
C8—C13—P2—C32	136.91 (19)	C31—C26—C27—C28	55.7 (3)
C12—C13—P2—C26	70.2 (2)	P2—C26—C27—C28	−176.7 (2)
C8—C13—P2—C26	−113.2 (2)	C26—C27—C28—C29	−55.5 (3)
C12—C13—P2—Pt1	−162.57 (19)	C27—C28—C29—C30	53.9 (4)
C8—C13—P2—Pt1	14.0 (2)	C28—C29—C30—C31	−53.1 (3)
C2—Si1—Pt1—P1	−16.37 (9)	C29—C30—C31—C26	53.7 (3)
C1—Si1—Pt1—P1	98.44 (11)	C27—C26—C31—C30	−55.1 (3)
C8—Si1—Pt1—P1	−141.38 (8)	P2—C26—C31—C30	174.6 (2)
C2—Si1—Pt1—P2	149.20 (9)	C13—P2—C32—C37	−71.31 (19)
C1—Si1—Pt1—P2	−95.99 (11)	C26—P2—C32—C37	176.08 (18)
C8—Si1—Pt1—P2	24.19 (8)	Pt1—P2—C32—C37	46.13 (19)
C20—P1—Pt1—Si1	−111.73 (10)	C13—P2—C32—C33	162.15 (18)
C7—P1—Pt1—Si1	10.95 (9)	C26—P2—C32—C33	49.5 (2)
C14—P1—Pt1—Si1	125.73 (9)	Pt1—P2—C32—C33	−80.41 (18)
C20—P1—Pt1—P2	−165.73 (11)	C37—C32—C33—C34	59.5 (3)
C7—P1—Pt1—P2	−43.05 (12)	P2—C32—C33—C34	−173.69 (17)
C14—P1—Pt1—P2	71.72 (11)	C32—C33—C34—C35	−58.3 (3)
C20—P1—Pt1—Cl1	66.92 (10)	C33—C34—C35—C36	55.5 (3)
C7—P1—Pt1—Cl1	−170.41 (8)	C34—C35—C36—C37	−52.8 (3)
C14—P1—Pt1—Cl1	−55.63 (8)	C35—C36—C37—C32	54.3 (3)
C13—P2—Pt1—Si1	−22.03 (8)	C33—C32—C37—C36	−58.1 (3)
C32—P2—Pt1—Si1	−136.12 (9)	P2—C32—C37—C36	172.88 (18)
